# Supplementary material for: Splice-Junction-Based Mapping of Alternative Isoforms in the Human Proteome
Source: Cell Rep. Author manuscript; Available in PMC 2020 Jan 15. (PMC6961840; doi:10.1016/j.celrep.2019.11.026)
Supplement: 2 [file NIHMS1546469-supplement-2.zip › DF1/PXD006675/LeftCoronaryArtery/LeftCoronaryArtery_15_BBS1_EGQSAPLLSAHVNMPGSEGLAAPNRPLNPE.pdf]

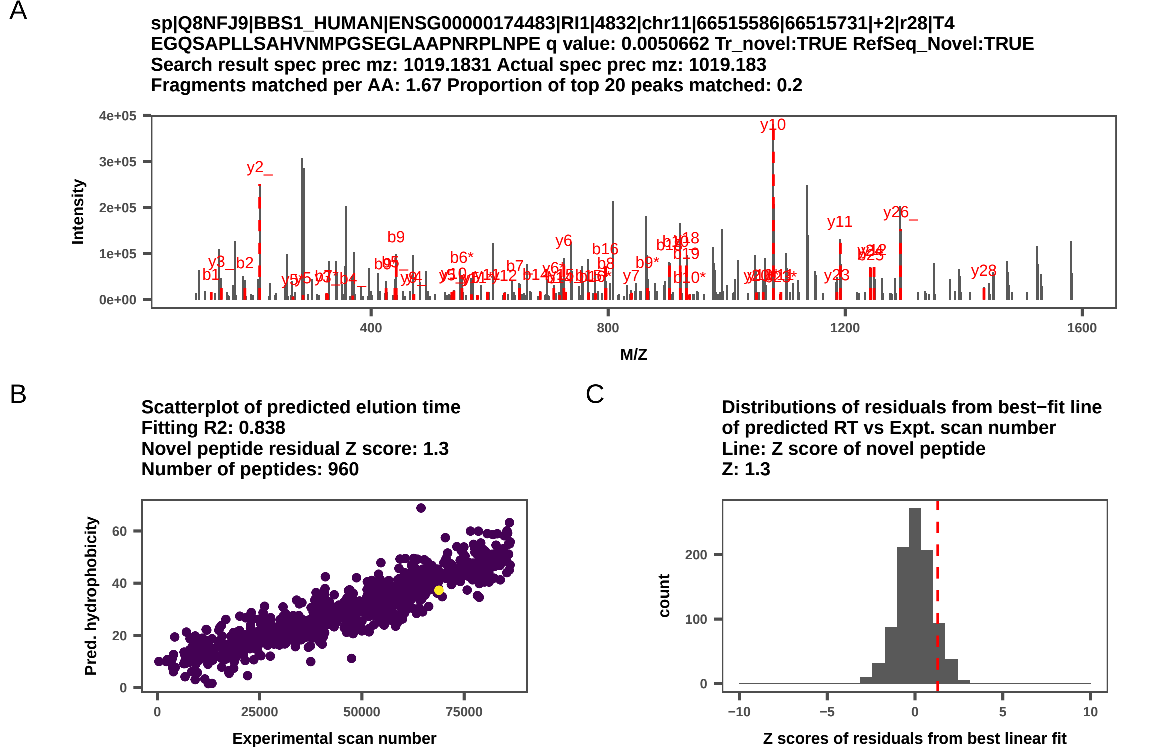
A
sp|Q8NFJ9|BBS1_HUMAN|ENSG00000174483|RI1|4832|chr11|66515586|66515731|+2|r28|T4
EGQSAPLLSAHVNMPGSEGLAAPNRPLNPE q value: 0.0050662 Tr_novel:TRUE RefSeq_Novel:TRUE
Search result spec prec mz: 1019.1831 Actual spec prec mz: 1019.183
Fragments matched per AA: 1.67 Proportion of top 20 peaks matched: 0.2
Intensity
4e+05
3e+05
2e+05
1e+05
0e+00
y2_
y10
y11
y26_
y28
b9
y6
b16
b6*
b7
y7
b9*
b19
b10*
y23
y3_
b2
b1
400
800
1200
1600
M/Z
B
Scatterplot of predicted elution time
Fitting R2: 0.838
Novel peptide residual Z score: 1.3
Number of peptides: 960
Pred. hydrophobicity
60
40
20
0
0
25000
50000
75000
Experimental scan number
C
Distributions of residuals from best−fit line
of predicted RT vs Expt. scan number
Line: Z score of novel peptide
Z: 1.3
count
200
100
0
−10
−5
0
5
10
Z scores of residuals from best linear fit
